# Effects of brief remote high ventilation breathwork with retention on mental health and wellbeing: a randomised placebo-controlled trial

**DOI:** 10.1038/s41598-024-64254-7

**Published:** 2024-07-23

**Authors:** Guy W. Fincham, Elissa Epel, Alessandro Colasanti, Clara Strauss, Kate Cavanagh

**Affiliations:** 1 School of Psychology, University of Sussex, Brighton, UK; 2 Brighton & Sussex Medical School, University of Sussex, Brighton, UK; 3 Present Address: Department of Psychiatry and Behavioral Sciences, University of California, San Francisco, USA; 4 Sussex Partnership NHS Foundation Trust, Worthing, UK

**Keywords:** Psychology, Human behaviour, Quality of life

## Abstract

High ventilation breathwork with retention (HVBR) has been growing in popularity over the past decade and might be beneficial for mental and physical health. However, little research has explored the potential therapeutic effects of brief, remotely delivered HVBR and the tolerability profile of this technique. Accordingly, we investigated the effects of a fully-automated HVBR protocol, along with its tolerability, when delivered remotely in a brief format. This study (NCT06064474) was the largest blinded randomised-controlled trial on HVBR to date in which 200 young, healthy adults balanced for gender were randomly allocated in blocks of 2 by remote software to 3 weeks of 20 min daily HVBR (fast breathing with long breath holds) or a placebo HVBR comparator (15 breaths/min with short breath holds). The trial was concealed as a ‘fast breathwork’ study wherein both intervention and comparator were masked, and only ~ 40% guessed their group assignment with no difference in accuracy between groups. Both groups reported analogous credibility and expectancy of benefit, subjective adherence, positive sentiment, along with short- and long-term tolerability. At post-intervention (primary timepoint) for stress level (primary outcome), we found no significant group × time interaction, *F*(1,180) = 1.98, *p* = 0.16, *η*_*p*_^2^ = 0.01, *d* = 0.21), nor main effect of group, (*F* = 0.35, *p* = 0.55, *η*_*p*_^2^ < 0.01) but we did find a significant main effect of time, (*F* = 13.0, *p* < 0.01, *η*_*p*_^2^ = 0.07). There was a significant improvement in stress pre-post-intervention in both groups, however there was no significant difference in such improvement between groups. In addition to stress at follow-up, we found no significant group x time interactions for secondary trait outcomes of anxiety, depression, mental wellbeing, and sleep-related impairment. This was also the case for state positive and negative affect after the first session of breathwork and at post-intervention. Brief remote HVBR therefore may not be more efficacious at improving mental health than a well-designed active comparator in otherwise healthy, young adults.

## Introduction

Volitional controlled respiration techniques (breathwork) may offer an accessible tool to combat adverse stress afflicting populations globally, with chronic stress affecting both mental and physical health at scale^1^. A recent meta-analysis of randomised controlled trials (RCTs) showed breathwork was connected to lower stress, anxiety and depression versus non-breathwork controls^2^. Over 75% of RCTs were primarily focused on slow breathwork, however preliminary data also suggest benefits of fast breathwork. For instance, Goldstein et al.^3^ reported larger improvements on subjective wellbeing measures such as stress, anxiety, depression, and sleep in an intervention comprising the fast-breathing technique called Sudarshan Kriya, compared to an active comparison in the form of a wellness workshop.

Fast breathwork has been proposed to provide a nonpharmacological alternative to psychedelics for therapeutic benefit (i.e., stress reduction) and inducing altered states of consciousness, and there has been an unprecedented surge in public/scientific interest in such ‘high ventilation breathwork’ (HVB) modalities^4^. The rubric of HVB may be defined as breathwork techniques where the rate and/or depth of ventilation is increased above the normal range, and we are using this umbrella label to respect and include the diverse scientific, spiritual, and religious communities which may use such practices. In addition to increased respiratory rate/depth, intensive HVB practices may include many other characteristics including (but not limited to): environmental context and cues conducive to contemplative practices, psychological priming and integration, group settings, sharing and discussion, meditative art, music, visualisation and ‘body work’ (i.e., physical touch)—see Fincham et al.^4^ for full review of various HVB practices. The vast majority of HVB sessions are performed in the presence of a trained breathwork facilitator(s) and the duration can vary dramatically, from a few minutes to a few hours.

One particularly accessible HVB style, which is frequently practised briefly (i.e., 10–20 min at a time) through remote digital/app-based means, incorporates hyperventilation (high ventilation)—usually around 30–40 deep fast breaths—with breath-holds (retention) frequently ranging anywhere from 1 to 3 min. This HVB with retention (HVBR) practice shares some similarities with Tibetan Tummo meditation and Yogic Pranayama but has been popularised in the West by the Wim Hof Method (WHM), primarily a combination of HVBR and cold exposure (performed separately)^4^. A landmark RCT showed for the first time that the autonomic nervous and innate immune systems could be voluntarily influenced via WHM training^5^. An immune challenge of bacterial endotoxin was administered to both novices who had undertaken a 10-day WHM program, and WHM-naïve controls who had not. Upon administration, the intervention participants practised HVBR and displayed reduced pro-inflammatory reactions, versus the inactive control group. Greater levels of adrenaline during HVBR were associated with larger and smaller anti-inflammatory and inflammatory responses, respectively.

Emerging research has also started exploring subjective effects of HVBR on mental health and wellbeing. Eight weeks of WHM training was associated with lower self-reported depression in comparison to an inactive control^6^ but this was an observational, non-randomised study limiting conclusions that can be drawn. On the other hand, a recent randomised trial showed two weeks of either the WHM (combination of daily cold shower and HVBR *separately*), solely HVBR, or cold exposure only were connected to lower subjective stress versus an inactive comparator, with the combination having the greatest effect^7^. However, the statistical analysis used was not explicit about whether interaction effects were significant, instead only showing post-hoc differences between groups in post-intervention levels of stress. Moreover, a recent RCT reported only 5 min/day of HVBR for four weeks being associated with improvements over time on state anxiety along with positive and negative affect, although this improvement was not significantly different from the control condition of mindfulness meditation for the same duration^8^.

While HVBR can provoke subacute anxiety, this may have therapeutic value with its effects being argued in terms of stress ‘exposure’ and ‘inoculation’. For instance, HVBR may allow individuals to tap into voluntarily produced, stressful experiential states (doses/bouts of unnatural hyperventilation and extended breath holding) which, through exposure over time, may lead to better adaptative mental health in the long run, i.e., regarding response to stress in normal life^4^. Though increasing stress seemingly mitigates the improved health claims of HVBR, it might be elucidated more clearly through the proposed mechanistic notion of hormesis, an adaptive response to states of moderate bodily stress^9^. Intense physical exercise, for example, can improve health by volitionally inducing a sympathetic response (stress) initially, subsequently followed by adaptation. Emphasis remains on the deliberateness of HVBR; reflexive hyperventilation is related to anxiety, but intentional hyperventilation can be therapeutic^8,10^.

Despite emerging research on HVBR having a positive impact on wellbeing, there is published evidence which does not necessarily support this narrative^11^. Indeed, findings are mixed^12^. Establishing the efficacy of HVBR from the current research landscape is further limited by the quality of trial design and study methods used. For instance, there are no placebo-controlled studies exploring effects of HVBR, making it difficult to establish whether interventions had specific active effects on mood beyond attention/expectation. Further, more research is needed to gauge the tolerability profile of fast breathwork in general^2^. Whilst the aforementioned meta-analysis on RCTs did not find negative effects directly attributed to breathwork, less than 25% of studies actively reported on this. However, contraindications range from cerebrovascular and cardiovascular conditions to epilepsy and panic disorder^4^. More general safety recommendations surrounding HVBR relate to only practising in a safe environment away from water and hard ground.

The evidence-based picture surrounding HVBR is ambiguous, warranting a well-designed study as a tool to add credibility to an uncertain field of findings currently in its infancy. Building upon a robustly designed slow breathwork study^13^, we planned to complete a RCT comparing brief (~ 20 min/day) remotely delivered HVBR for three weeks to a ‘placebo’ HVBR comparator (paced breathing at 15 breaths/min with short breath holds). The metric of 15b/min is within the range of normal, healthy resting respiration of 12–16b/min^14^. Fundamentally, for the intervention, we extracted a key component shared by various HVB practices, i.e., fast breathing in a ‘conscious connected’ style (no pauses between inhales and exhales) and adapted this so that it was brief and delivered remotely. Accordingly, this was a ‘reductionist’ approach compared to some ‘real world’ HVB practices. Seeing as tolerability was an outcome of particular interest and owing to very little research on HVBR to date, this RCT was conducted within young, otherwise healthy adults.

The primary research question was whether HVBR would lead to improved perceived stress level (primary outcome) in comparison to the active comparison in a general population adult sample at post-intervention (primary timepoint). We thus essentially examined whether the HVBR component of a technique like the WHM—when delivered remotely—was able to improve stress compared to another breathing-focused practice matched for time and attention but at a breath rate and retention duration that would presumably not alter the autonomic nervous system in a dramatic way. Secondary outcomes included anxiety, depression, mental wellbeing, sleep-related impairment, positive and negative affect, credibility and expectancy of the breathwork, short- and long-term side effects from the breathwork, and self-reported adherence. Other additional outcomes included overall experience of, and sentiment towards, the breathwork and whether participants could guess which condition they were allocated to.

## Materials and methods

### Randomisation and blinding

Participants enrolled through the research platform Prolific (prolific.com) but completed questionnaires via integration of the survey platform Qualtrics (qualtrics.com). Accordingly, this was a participant-blinded, superiority RCT with the assessors blinded to randomisation and data collection (research team were not present for data collection as this was self-completed by participants remotely through Qualtrics). After completing the baseline survey, participants were automatically randomised, via Qualtrics using block randomisation (1:1), to receive either the intervention or active comparator. Participants were blinded to their randomly assigned intervention (concealment)—all saw that the study was referred to as the “Sussex Fast Breathwork Study” and the breathwork technique in both groups was referred to as “Rhythmic Breathing” in an attempt to mask the intervention being used.

### Intervention

Participants in the intervention group were randomised to a guided audio of HVBR pre-recorded by a trained breathwork facilitator from the organisation Othership (othership.us) for ~ 20 min/day over 21 days. This was delivered remotely online through private, downloadable audio link. Participants were instructed to ensure they were in a comfortable, quiet and safe environment, lying down with headphones in and eyes closed or covered. They were informed they would be doing four rounds of upregulated (fast) breathing with holds on empty for each round, and as the rounds progressed so too would the breathing and breath holds (see Table 1 for an overview). It was also clearly stated that if at any point participants needed to breathe during the retentions this was absolutely fine. The session comprised evocative music and four rounds of hyperventilation progressively increasing in intensity (up to ~ 60b/min) with four separate retentions on empty (following an exhale), gradually increasing in length from ~ 45 s up to ~ 90 s. After each retention, participants were instructed to take a deep breath in and hold for ~ 10secs before releasing. This protocol is similar to the WHM breathing but more standardised in retention rates.

**Table 1:** Overview of intervention and control group instructions, showing the respiratory rate per minute, length of each round and the average retention duration in seconds.

	Round 1		Round 2		Round 3		Round 4	
	Breathing	Retention	Breathing	Retention	Breathing	Retention	Breathing	Retention
Intervention	Starts at 15 b/min increasing to 30 b/min (total: ~ 90 s)	~ 45 s	Starts at 15 b/min increasing to 30 b/min, & then 60 b/min (~ 120 s)	~ 60 s	Starts at 15 b/min increasing to 30 b/min, & then 60 b/min (~ 150 s)	~ 75 s	Starts at 30 b/min increasing to 60 b/min (~ 150 s)	~ 90 s
Control	15 b/min (total: ~ 125 s)	~ 10 s	15 b/min (~ 165 s)	~ 15 s	15 b/min (~ 205 s)	~ 20 s	15 b/min (~ 215 s)	~ 25 s

In both groups every retention was performed after taking a big inhale before exhaling and holding ‘on empty’. After each retention participants were then instructed to take a deep breath in and hold for ~ 10secs before releasing.

In order to control for as much variation as possible in breathing pattern, a conscious connected breathing (CCB) rhythm was recommended—actively filling lungs on the inhale and passively letting the air go on the exhale without any pausing between them^15^. Moreover, to remove the potential confound of nasal breathing conferring benefit^16–18^, mouth breathing was advised. After the last round was complete, participants were invited to take a moment of introspection and tune into how they were feeling. The average number of HVBR rounds used is typically 3–4 ^6,7^, thus the duration was considered a reasonable commitment for participants, with one study suggesting benefit can be derived from only 5 min/day of HVBR^8^.

### Comparison

Participants in the active comparator were randomised to a guided audio of ‘placebo’ HVBR pre-recorded by the same breathwork facilitator for ~ 20 min/day over three weeks (see Table 1 for an overview). This audio comprised evocative music and four rounds of paced breathing at 15b/min in a conscious connected style (no pauses between inhale:exhale) with four separate (shorter) retentions, increasing in length from ~ 10 s up to ~ 25 s. It was very similar to the intervention in both content and nature, but it was not possible to fully match the script and music of the comparator to the intervention owing to the complexity of the practices; nonetheless best attempts were made to make them as comparable as possible (supplement contains example links to recordings). Again, after each retention, instructions were to take a deep breath in and hold for ~ 10secs before releasing. After the last round, participants were given the same introspective instructions during the period of integration. The metric of 15b/min is in line with the resting respiratory rate ranging from 12 to 16b/min^14^, and we believed that this upper bound of 15b/min—paired with mouth breathing—could create the illusion of fast breathwork. Moreover, the same language in the intervention was used to describe the breathing in the active comparison to try and conceal the HVBR control.

### Procedure

Remote delivery was through audio links, and daily reminders were sent on Prolific to practise and keep a record of whether one had practised each day. Participants provided informed consent and were paid £9/hr (recommended by Prolific) to complete the questionnaires. The primary timepoint and primary outcome were post-intervention and level of stress, respectively. Trait mental health measures of stress level along with anxiety, depression, wellbeing, and sleep-related impairment (secondary outcomes) were measured at three timepoints; baseline, post-intervention and follow-up (before, after, and three weeks after the intervention). Subjective credibility and expectancy of the breathwork for both groups were measured immediately after participants practised their first breathwork session. In addition, state mental health measures of positive and negative affect were completed at baseline, immediately after the first breathwork session, and post-intervention. The final secondary outcomes, measured at post-intervention, were: short-term and long-term negative effects and subjective adherence to the breathwork protocol.

### Outcome measures

The Depression Anxiety Stress Scale-21 (DASS-21)^19^ subscales were used to measure the primary outcome of stress along with secondary outcomes of anxiety and depression. This is a psychometrically well-validated measure^20^ and Cronbach’s alphas (*α*) showed high to very high internal consistency at baseline across stress (7 items; *α* = 0.87), anxiety (7 items; *α* = 0.82), and depression (7 items; *α* = 0.92). The response frame is “over the past week” with a score range of 0–21 for each subscale; scores are then converted to the longer form DASS-42 total by multiplying by two. For example, item one (stress) reads “I found it hard to wind down”, scored as 0 (“Did not apply to me at all”), 1 (“Applied to me to some degree, or some of the time”), 2 (“Applied to me to a considerable degree or a good part of time”), or 3 (“Applied to me very much or most of the time”). Higher scores denoted worse outcomes, and normal stress is scored 0–14. Item two (anxiety) and item three (depression) read “I was aware of dryness of my mouth” and “I couldn’t seem to experience any positive feeling at all”, respectively, with the same scoring from 0 to 3 for each item.

The other trait measures were mental wellbeing (using the Short Warwick-Edinburgh Mental Wellbeing Scale; SWEMWBS)^21^ and sleep-related impairment (PROMIS Item Bank v. 1.0—Sleep-Related Impairment—Short Form 4a; PROMIS-4a)^22^. The SWEMWBS is psychometrically well-validated^23^ and showed high internal consistency at baseline (7 items; *α* = 0.89). Its response frame is over the past two weeks. For instance, item one reads “I’ve been feeling optimistic about the future”, scored from 1 (“None of the time”) to 5 (“All of the time”). Total raw scores are transformed into metric scores using the SWEMWBS conversion table (at warwick.ac.uk). The final score range is 7–35 with lower scores denoting worse outcomes. The PROMIS-4a contains well-validated items^24,25^ and displayed very high internal consistency at baseline (4 items; *α* = 0.94). Its response frame is over the past week. Item one reads “I had a hard time getting things done because I was sleepy”, scored from 1 (“Not at all”) to 5 (“Very much”). Total raw scores are converted using a T-score transformation (at healthmeasures.net). The final range is 36.2–77.7, with higher scores indicating worse outcomes.

To gauge state change arising from the breathwork (if any), the state outcomes of positive and negative affect were measured via the subscales of the Positive and Negative Affect Schedule (PANAS-20)^26^. The PANAS-20 is well-validated^27,28^ and showed very high internal consistency at baseline for both positive affect (10 items; *α* = 0.93) and negative affect (10 items; *α* = 0.91). The response frame is in the moment (“right now”) and has a score range of 10–50 for each subscale. For example, item one (positive affect) and item two (negative affect) respectively read “Interested” and “Distressed”, scored from 1 (“Very slightly or not at all”) to 5 (“Extremely”). Higher scores on positive and negative affect denoted better and worse outcomes, respectively.

Credibility and expectancy of the breathwork were measured by the Credibility/Expectancy Questionnaire (CEQ-6)^29^. This was used to assess whether the intervention and comparator were viewed as proportionately credible warranting equivalent expectation of therapeutic value. The CEQ-6 is psychometrically well-validated^29,30^ and showed very high internal consistency at baseline among credibility (3 items; *α* = 0.91) and expectancy (3 items; *α* = 0.91). The measure has two sets of questions for credibility and expectancy of said course/therapy (in our case breathwork). For instance, item one (credibility) reads “At this point, how logical does the breathwork offered to you seem?”, scored from 1 (“Not at all logical”) to 9 (“Very logical”), and item four (expectancy) reads “By the end of the breathwork period, how much improvement in your mental health and wellbeing do you think will occur?”, scored from 0 to 100%. Each credibility item is scored 1–9, whilst expectancy contains one item scored 1–9 and two items scored 0–100%. To standardise the latter, scores for expectancy are converted to *z*-scores. Higher scores implied higher levels of perceived credibility/expectancy of the breathwork.

Subjective adherence was simply measured via the number of times out of the 21 sessions assigned participants stated they practised the breathwork. The remaining secondary outcomes of self-reported tolerability were divided into short-term and long-term negative effects of the breathwork. Participants indicated the extent to which they agreed or disagreed (from “Strongly agree” to “Strongly disagree”) with experiencing any unpleasant/unwanted short-term effects from the breathwork. The same applied for long-term and regarded experiencing any lasting bad effects from the breathwork.

Other outcomes included general sentiment towards the breathwork (from “Strongly positive” to “Strongly negative”) and short optional, open-ended questions on overall experiences of the protocol (to describe it as briefly as possible in one or more words) and/or study period. Finally, to tentatively infer if blinding was successful, participants were informed at the end of the follow-up survey that they were either in an intervention or control group and were asked to guess which they were allocated to.

### Statistical analyses

The software *R* (v4.3.2) was used for the primary data analysis (intention-to-treat), with the primary timepoint, primary outcome and alpha level being post-intervention, subjective stress and *p* < 0.05, respectively. As seen above, internal reliability of baseline scales was assessed using Cronbach’s alphas (*α*). Mixed repeated-measures analysis of variance was conducted to determine group x time effects for the trait and state scale outcomes measured across multiple timepoints. Simple contrasts using baseline as the comparator, in addition to within-group t-tests (applying a Bonferroni correction), would follow up significant (*p* < 0.05) group × time effects, if any. Regarding missing data, a sensitivity analysis was conducted using 30 imputed datasets, comprising any study variable that predicted missingness or outcomes. The secondary data analysis (per-protocol) included only those participants who self-reported practising breathwork for 10 or more of the allocated 21 sessions. Independent-tests compared CEQ-6 scores and subjective adherence between the intervention and control. Moreover, to take into account the use of multiple correlational analyses via changing the significance level to *p* < 0.01, such scores and adherence were correlated with changes in the trait and state scale scores between baseline and post-intervention, to enable expectation and dose-response analyses.

### Ethics approval

This study was approved by the Sciences and Technology Cross-Schools Research Ethics Committee (University of Sussex; ref. ER/GF221/5), preregistered with ClinicalTrials.gov: NCT06064474 (03/10/2023), and conducted in accordance with the tenets of the Declaration of Helsinki. It was remotely conducted via the Prolific research platform, and all participants provided informed consent.

### Consent to participants

Due to the nature and novelty of the HVBR intervention and, since tolerability of the fast breathwork was an outcome of interest, self-assessed inclusion criteria were extensive: 18–39, fluent in English/living in UK, approval rate of 98% or higher and over 20 previous submissions on Prolific (i.e., they had been part of Prolific studies before, suggested by the platform for longitudinal studies with several timepoints), access to headphones, comfortable with faster breathing and breath holding, willing to only practise breathwork in a safe environment and always away from water, hard ground, large meals and bedtime.

Self-assessed exclusion criteria included: history of hypotension or hypertension, respiratory or cardiovascular problems, fainting or syncope, epilepsy or seizures, panic disorder or panic attacks, cerebral aneurysm, problems with prior breathwork sessions, pregnancy (and possibility one might be pregnant, trying to get pregnant, or are breastfeeding), any problems which affect one’s ability to pace breathing, breathlessness, bradypnea or tachypnoea, other physical/mental health conditions/current life events impairing or affecting one’s ability to engage in activities involving breath control, and finally taking any medication (other than the contraceptive pill) such as those to reduce blood pressure, beta blockers, antidepressants, anxiolytics or any other psychotropic medications.

We recruited 200 participants via the ‘balanced sample’ option on Prolific so as to distribute the study evenly across gender, which allowed greater power than needed: A medium effect size of 0.50, statistical power of 0.80, and significance level of 0.05, respectively, required a sample size of 128. PsyDAO, a decentralised autonomous organisation supporting research at the intersection of psychedelics and mental health, provided funding for participant payments for a sample of 200, so this was the largest possible, and also allowed for potential attrition. The two RCTs focusing on HVBR and mental health had final samples of *N* = 108 (four groups; HVBR condition,* n* = 33)^8^ and *N* = 86 participants (4 groups; HVBR condition, *n* = 20)^7^. The former study was not adequately powered statistically to compare HVBR with the other two breathwork techniques (only the combined effects of three breathwork conditions to a meditation condition), and the latter stated actually requiring a sample size of 100, however it detected a medium-large significant effect size for the WHM.

## Results

**Figure 1:**
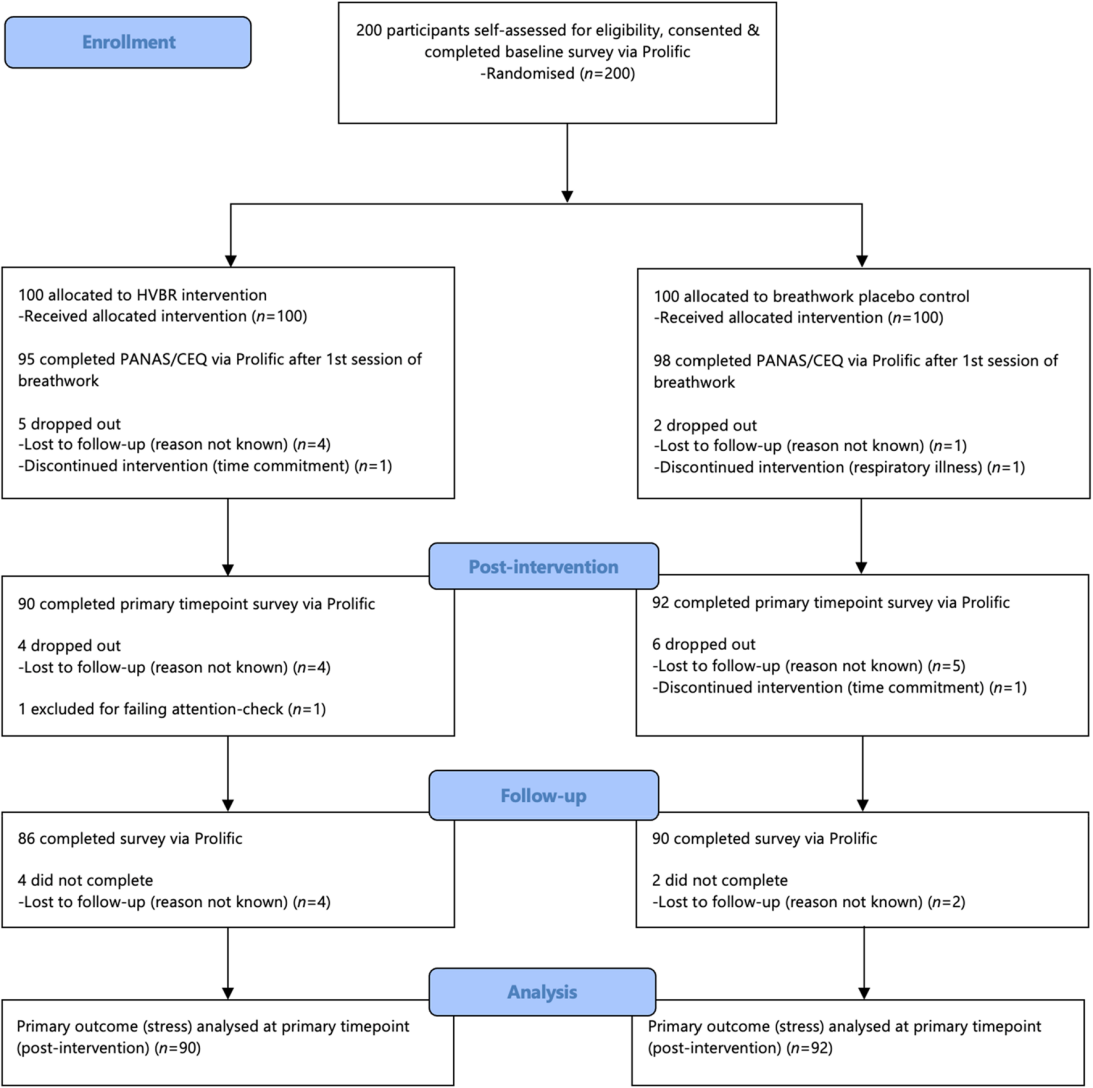
Study participant flow.

Study recruitment via Prolific began and was completed on October 13, 2023. In line with Fig. 1, (CONSORT flow diagram), 200 participants were recruited and 182 completed the primary outcome measure (stress) at the primary timepoint (post-intervention). Table 2 displays the baseline demographics and measures (M ± SD scores)—there were no significant differences across these. A balanced sample in terms of age and gender was recruited, and most participants were of white ethnicity. There were no significant differences between the 18 study non-completers and 182 completers (those who completed the primary outcome at primary timepoint) apart from positive affect which was significantly higher in the completers. Table 3 shows the mental health and wellbeing outcome measure scores at each timepoint.

**Table 2:** Baseline demographics and measures. *Any other ethnic group or prefer not to say, Mixed = two or more ethnic groups.

Characteristic	Group		
	Intervention (*n* = 100)	Comparator (*n* = 100)	Test statistic *(p)*
Gender (*n*)			*Χ*^2^_2_ = 0.02 (0.89)
Female	51	50	
Male	49	50	
Age (M ± SD)	31.50 ± 5.13	31.67 ± 4.87	*t* = 0.24 (0.80)
Ethnicity (*n*)			*χ*^2^_5_ = 5.34 (0.38)
White	78	80	
Black	7	8	
Asian	11	5	
Mixed	2	6	
Other*	2	1	
DASS (M ± SD)	29.12 ± 21.29	25.36 ± 19.51	*t* = 1.30 (0.19)
Stress	12.62 ± 7.55	11.96 ± 8.14	*t* = 0.59 (0.55)
Anxiety	6.44 ± 6.76	5.02 ± 5.39	*t* = 1.64 (0.10)
Depression	10.06 ± 9.59	8.38 ± 8.55	*t* = 1.31 (0.19)
SWEMWBS	22.21 ± 4.48	21.48 ± 4.09	*t* = 1.20 (0.23)
PROMIS	54.39 ± 9.79	54.70 ± 10.23	*t* = -0.22 (0.83)
PANAS	44.43 ± 9.92	42.91 ± 8.86	*t* = 1.14 (0.25)
Positive affect	30.07 ± 8.86	29.71 ± 8.64	*t* = 0.29 (0.77)
Negative affect	14.36 ± 6.36	13.20 ± 4.51	*t* = 1.49 (0.14)

**Table 3:** Scores for all mental health and wellbeing measures.

	Baseline		After 1st session		Post-intervention		Follow-up	
	*n*	Mean (SD)	*n*	Mean (SD)	*n*	Mean (SD)	*n*	Mean (SD)
Positive affect								
Intervention	100	30.07 (8.86)	95	30.33 (9.28)	90	31.09 (8.45)		
Control	100	29.71 (8.64)	98	29.26 (9.15)	92	30.23 (9.12)		
Negative affect								
Intervention	100	14.36 (6.36)	95	12.71 (4.69)	90	14.27 (5.76)		
Control	100	13.20 (4.51)	98	11.94 (3.33)	92	13.78 (5.53)		
Stress level								
Intervention	100	12.62 (7.55)			90	10.31 (7.11)	86	12.14 (7.96)
Control	100	11.96 (8.14)			92	11.17 (8.34)	90	11.71 (7.52)
Anxiety level								
Intervention	100	6.44 (6.75)			90	5.62 (6.46)	86	6.77 (7.50)
Control	100	5.02 (5.39)			92	5.65 (6.64)	90	5.84 (6.01)
Depression level								
Intervention	100	10.06 (9.59)			90	7.16 (7.66)	86	8.40 (8.86)
Control	100	8.38 (8.55)			92	7.98 (8.95)	90	8.73 (9.71)
Mental wellbeing								
Intervention	100	22.21 (4.48)			90	22.74 (4.03)	86	23.00 (4.24)
Control	100	21.48 (4.09)			92	22.17 (4.55)	90	22.23 (4.46)
Sleep-related impairment								
Intervention	100	54.39 (9.79)			90	52.58 (9.80)	86	52.90 (10.71)
Control	100	54.70 (10.23)			92	53.07 (9.97)	90	54.19 (10.90)

Primary outcome and primary timepoint are stress and post-intervention, respectively. Positive and negative affect measured after first session of breathwork to gauge state change, if any. Stress, anxiety, depression, mental wellbeing and sleep-related impairment measured at follow-up to gauge trait change, if any.

### Credibility, expectancy and state affect

193 participants completed the CEQ and PANAS after practising their first session of breathwork. Independent t-tests revealed no significant differences on credibility scores (M ± SD) between the intervention (17.69 ± 5.33) and comparator (17.18 ± 5.32), *t*(191) = 0.67, *p* = 0.51), nor any significant differences on expectancy *z*-scores (M ± SD) between the intervention (− 0.03 ± 0.91) and comparator (− 0.03 ± 0.93), (*t*(191) = 0.51, *p* = 0.61), inferring no differences in credibility/expectancy between the conditions. Again, adjusting alpha to account for multiple correlational analyses, there were no significant correlations between these two measures and changes in the primary and secondary outcome scale scores pre-post intervention in both conditions, apart from a weak-moderate association between expectancy and negative affect in the intervention group (*n* = 95) *τ*_*B*_ = −0.22, *p* < 0.01.

After the first session of breathwork, we found no significant group x time interactions for positive and negative affect, (*F*(1,191) = 0.07, *p* = 0.79, *η*_*p*_^2^ < 0.01; *F* < 0.01, *p* = 0.96, *η*_*p*_^2^ < 0.01, respectively). We found a group main effect for positive affect and negative affect, (*F* = 5.68, *p* = 0.02 *η*_*p*_^2^ = 0.03; *F* = 11.3, *p* < 0.01, *η*_*p*_^2^ = 0.15, respectively), which were both slightly greater in the intervention group. We found no time main effect for positive affect, but we did for negative affect (*F* = 1.56, *p* = 0.21, *η*_*p*_^2^ < 0.01; *F* = 19.4, *p* < 0.01, *η*_*p*_^2^ = 0.09, respectively). Accordingly, there was no state improvement for positive affect from baseline to after the first breathwork session across both conditions, however there was a state change and significant improvement in negative affect for both groups. Nonetheless there were no differences between the groups on such improvement.

### Adherence to protocol

Independent t-tests revealed no significant differences on subjective adherence (M ± SD sessions practised) between the intervention (14.08 ± 6.34) and comparator (13.23 ± 5.90), *t*(180) = 0.94, *p* = 0.35). Where a participant provided a range (i.e., “18–19 days” and “13–14 times”, respectively), the lowest/minimum values were used (i.e., 18 and 13 sessions, respectively) to provide the most conservative estimate. Adjusting alpha for multiple analyses, there were no significant correlations between adherence and changes in the outcome scale scores pre-post intervention in both conditions, apart from a weak-moderate association with stress in the comparator (*n* = 98) *τ*_*B*_ =  − 0.23, *p* < 0.01.

### Short-term tolerability

In the intervention group, 60 (66%) participants strongly disagreed with experiencing any unpleasant/unwanted short-term effects from the breathwork, 15 (16%) slightly disagreed, 8 (9%) were neutral, 6 (7%) slightly agreed, and 2 (2%) strongly agreed. In the comparator, 58 (64%) participants strongly disagreed with experiencing any unpleasant/unwanted short-term effects from the breathwork, 20 (22%) slightly disagreed, 5 (5%) were neutral, 8 (9%) slightly agreed, and no one strongly agreed. A Chi-squared test revealed no significant differences between the two groups, *χ*^2^_5_ = 5.26 (0.39), suggesting similar short-term tolerability across groups.

The very small number of reasons for agreeing with the statement of experiencing negative, unpleasant/unwanted short-term effects in the intervention group included: light-headedness, dizziness, sleepiness, initial negative feelings of sadness (but which gradually improved), and tingly sensations paired with inability to use fingers (i.e., tetany). Reasons not directly attributable to the intervention included it being time-consuming (due to other commitments) and stress regarding remembering to practise. In the comparator, reasons, all of which were directly attributable to the HVBR comparator, included: light-headedness, dizziness, stress in relation to the music, tension in chest, and the breathing pace being slightly too fast thereby causing some mild anxiety.

### Long-term tolerability

In the intervention group, 72 (79%) participants strongly disagreed with experiencing any lasting adverse effects from the breathwork, 12 (13%) slightly disagreed, 7 (8%) were neutral, and no one slightly nor strongly agreed. In the comparator, 75 (82%) participants strongly disagreed with experiencing any lasting adverse effects from the breathwork, 15 (16%) slightly disagreed, 1 (1%) was neutral, and no one slightly nor strongly agreed. There was no significant difference between the two groups, *χ*^2^_4_ = 6.51 (0.16), suggesting similar long-term tolerability.

### Blinding to breathwork

When participants were asked at follow-up to guess which group they were in, in the intervention group 36 (42%) guessed correctly, 33 (38%) incorrectly, and 17 (20%) were unsure. In the comparator, 30 (33%) guessed correctly, 36 (40%) guessed incorrectly, and 24 (27%) were unsure. There was no significant difference between the two groups, *χ*^2^_3_ = 2.54 (0.47), tentatively suggesting masking/concealment of, and blinding to, the practices was successful.

### Experience of breathwork

In terms of overall experience of the breathwork protocol, in the intervention group, 32 (35%) reported strongly positive sentiment towards the breathwork, 44 (48%) slightly positive, 10 (11%) neutral, 3 (3%) slightly negative, and 2 (2%) strongly negative. In the comparator, 22 (24%) reported strongly positive sentiment, 44 (48%) slightly positive, 18 (20%) neutral, 5 (5%) slightly negative, and 2 (2%) strongly negative. There was no significant difference between the two conditions, *χ*^2^_5_ = 4.91 (0.43), suggesting similar sentiment among groups.

### Primary outcome stress level

**Figure 2:**
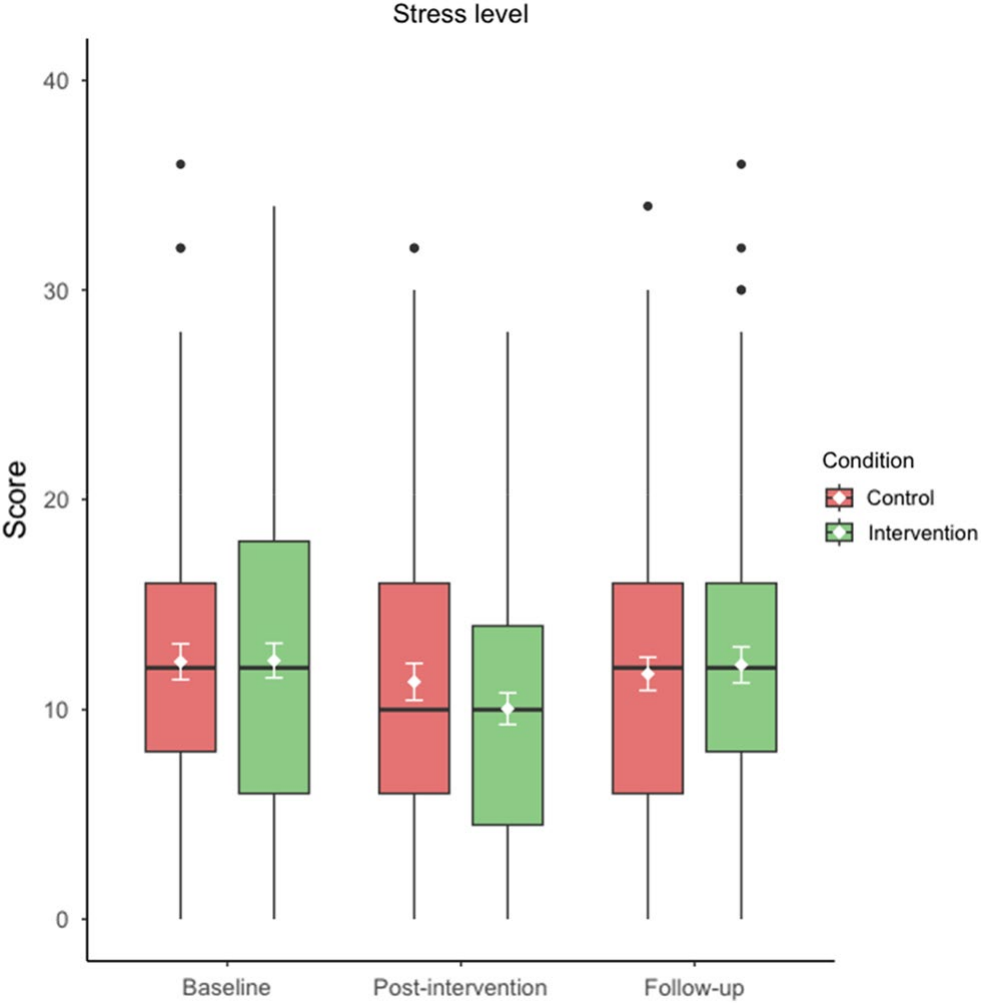
Score distribution for primary outcome stress across all timepoints (pre-post-follow-up) for both intervention (green) and control (red) conditions. Means (± 95% CI error bars) in white and medians are middle lines within boxes, with higher scores inferring greater stress (score range 0–42). Boxplots also display quartiles and black circles represent outliers. Primary timepoint is post-intervention. Figure produced using *R* (version 4.3.2).

At post-intervention (primary timepoint for primary outcome) we did not find a significant group x time interaction for stress levels, (*F*(1,180) = 1.98, *p* = 0.16, *η*_*p*_^2^ = 0.01, *d* = 0.21) nor main effect of group, (*F* = 0.35, *p* = 0.55, *η*_*p*_^2^ < 0.01) but we did find a significant main effect of time, (*F* = 13, *p* < 0.01, *η*_*p*_^2^ = 0.07). In line with Table 3 and Fig. 2, there was a significant improvement in stress scores pre-post-intervention in both conditions, however there was no significant difference in such improvement across conditions. When including the follow-up timepoint in addition to baseline and post-intervention (pre-post-follow-up), we did not find a significant interaction, (*F*(1,174) = 0.16 *p* = 0.69, *η*_*p*_^2^ < 0.01) nor main effect of group or time, (*F* = 0.33, *p* = 0.57, *η*_*p*_^2^ < 0.01; *F* = 0.74, *p* = 0.39, *η*_*p*_^2^ < 0.01, respectively). Consistent with Table 3 and Fig. 2, there was no improvement in stress scores from baseline to follow-up in both conditions, with no differences across conditions. A sensitivity analysis comprising multiple imputation (30 datasets) of the very low amount of missing data at post-follow-up replicated these non-significant findings overall. The per-protocol analysis also replicated these non-significant findings for participants who reported practising 10 or more sessions.

### Secondary mental health and wellbeing outcomes

Scores for the secondary mental health, wellbeing and sleep-related impairment outcome scales are shown in Table 3 and supplement Figs. S1–S6.

### Positive and negative affect

At post-intervention, we found no significant group x time interactions for positive and negative affect, (*F*(1,180) = 0.03, *p* = 0.86, *η*_*p*_^2^ < 0.01; *F* = 0.09, *p* = 0.76, *η*_*p*_^2^ < 0.01, respectively), nor main time effects, (*F* = 0.311, *p* = 0.58, *η*_*p*_^2^ < 0.01; *F* = 0.58, *p* = 0.45, *η*_*p*_^2^ < 0.01, respectively), and group main effects, (*F* = 5.91, *p* = 0.02, *η*_*p*_^2^ = 0.03; *F* = 4.50, *p* = 0.04, *η*_*p*_^2^ = 0.02, respectively). Applying a Bonferroni-correction rendered the latter p-values non-significant. Accordingly, there was no improvement from baseline to post-intervention on these state measures and no difference between groups. Per-protocol analysis replicated these non-significant findings, in addition to no group main effects.

### Anxiety level

We found no significant group x time interaction for anxiety, (*F*(1,174) = 0.02, *p* = 0.88, *η*_*p*_^2^ < 0.01), nor group and time main effects, (*F* = 1.43, *p* = 0.23, *η*_*p*_^2^ < 0.01; *F* = 1.68, *p* = 0.20, *η*_*p*_^2^ < 0.01, respectively). Per-protocol analysis replicated these findings.

### Depression level

We found no significant group × time interaction for depression, (*F*(1,174) = 0.68, *p* = 0.44, *η*_*p*_^2^ < 0.01), nor group and time main effects, (*F* = 0.40, *p* = 0.53, *η*_*p*_^2^ < 0.01; *F* = 0.26, *p* = 0.61, *η*_*p*_^2^ < 0.01, respectively). Per-protocol analysis replicated these findings.

### Mental wellbeing

We found no significant group x time interaction for wellbeing, (*F*(1,174) = 0.61, *p* = 0.41, *η*_*p*_^2^ < 0.01). However, we did find a significant group and time main effect, (*F* = 9.66, *p* < 0.01, *η*_*p*_^2^ = 0.05; *F* = 5.37, *p* = 0.02, *η*_*p*_^2^ = 0.03, respectively). There was a significant improvement in mental wellbeing from baseline to follow-up in both groups but there was no difference between groups on the magnitude of this improvement. Per-protocol analysis replicated these findings.

### Sleep-related impairment

We found no significant group x time interaction for sleep-related impairment, (*F*(1,174) = 0.01, *p* = 0.91, *η*_*p*_^2^ < 0.01), nor group and time main effects, (*F* = 2.31, *p* = 0.13, *η*_*p*_^2^ < 0.01; *F* = 0.94, *p* = 0.33, *η*_*p*_^2^ < 0.01, respectively). Per-protocol analysis replicated these findings.

## Discussion

### Findings summarised

Our study offers robust insight into the impact of brief remote HVBR on psychological stress, mental health and wellbeing. We compared the subjective effects of three weeks of digitally administered HVBR and a ‘placebo’ HVBR (both offered for 20 min daily) on levels of stress (primary outcome), anxiety, depression, mental wellbeing, and sleep-related impairment, along with state measures of positive and negative affect. Participants were blind to the hypothesised direction of effects and assessors were not present during assessments. The study recruited a large sample (*N* = 200) of young adults (18–39), equally balanced for gender. The primary question was whether HVBR led to improved stress levels compared to the active control in a general, otherwise healthy population sample at post-intervention. In other words, using a reductionistic approach, we were testing whether brief cyclic hyperventilation with breath holds, done daily for a few weeks, had a significant effect. The results of the RCT did not show this. Our findings parsimoniously indicate that brief remote HVBR, in the form of the current study, was not more efficacious in lowering stress levels compared to the active breath-focused comparator, with this finding replicated for the secondary outcomes.

Furthermore, along with stress levels at longer term follow-up (a further three weeks post primary timepoint), there were no significant between-group differences in changes over time (from baseline to post-intervention to follow-up) for the secondary research questions of whether brief remote HVBR led to improved anxiety, depression, mental wellbeing and sleep-related impairment levels relative to the comparator. There were also no between-group differences in changes in positive and negative affect from immediately after the first session of breathwork to post-intervention. Consistent with the above, we managed to create a HVBR comparator that yielded equal credibility and expectation of benefit (according to CEQ scores), adherence (13–14 sessions in each group) and sentiment. Around 75% in each group reported a positive overall experience of the breathwork. Therefore, unsurprisingly, there were no differences between the groups in terms of blinding to the breathwork. In line with this robust study design, findings may be best interpreted as true null effects, however nuanced detail is provided in the following sections.

### Findings contextualised

Given a broad array of positive anecdotal reports regarding HVBR and recent studies pointing towards beneficial effects of HVBR pertaining to mental health and wellbeing^6–8^, our results are somewhat surprising. However, similar to our study on slow-paced breathwork^13^, it may be the case that when a more rigorous study design is applied to HVBR, including a robust, equally credible active comparator with equal expectation of benefit and a larger sample size, its effects are no longer apparent. From an alternative perspective and interpretation of the data, it could be the case that when we remove other elements of HVB, and control for a conscious connected style of breathing, there is no differential effect associated to fast breathing when delivered as a brief and remote intervention. This may have eliminated the contribution of two purported key ingredients of more powerful HVB techniques rooted in ‘spiritual’ and ‘mystical’ traditions: (1) psychological integration and (2) positive psychedelic-like subjective experiences. Nonetheless, a recent elegant preprint showed 14 participants, who practised remote HVBR for 20–60 min/day over four weeks, reported psychedelic-like phenomenological substates such as high bliss^31^. This could mean remote HVBR might be associated with psychedelic-like experiences but not necessarily measurable benefits in stress reduction.

Importantly, we are not unique in finding null effects for HVBR. A very recent study^32^ under peer-review found there was no difference between a 3-week intervention in the form of the WHM (daily 15 min HVBR, plus a cold shower) and a control condition involving slow breathing and warm showers on affective traits. In fact, they were both equally effective at lowering subjective stress, anxiety and depression in a sample of over 80 women with high depressive symptoms. Another recent RCT of 42 male participants using HVBR (plus cold water exposure and meditation, i.e., WHM-style) showed just over two weeks of daily practise, in fact, did not result in improvements on subjective measures compared to the control group including levels of stress, positive affect, negative affect, and vitality (state and trait), nor objective cardiovascular-related outcomes^11^. Nonetheless, it is important to note that both ours and Ketelhut et al.’s samples were young, otherwise healthy adults. This demographic may simply not derive as much (if any) benefit from brief HVBR as compared to, for example, a subset of the population with poor health. Indeed, more specifically, emerging literature has suggested that research focus on the WHM should shift from healthy participants and move in the direction of rigorously exploring the methods’ effects in non-healthy populations with inflammatory conditions, owing to its promising use case in inflammatory responses^12^.

Since very little research on HVBR has been conducted to date, we deemed it necessary to recruit a healthy sample, as we were also particularly interested in the tolerability of this technique. There were no significant differences between the intervention and comparator in terms of short- and long-term side effects, suggesting similar tolerability across groups. No participants agreed with the statement of experiencing any lasting bad effects from the breathwork, suggesting a high tolerability profile over three weeks. Only 9% in each group agreed with the statement of experiencing any unpleasant/unwanted short-term effects from the breathwork. A very small number of acute side effects were reported and, interestingly, they were similar in both groups.

Though the preliminary tolerability data are encouraging, it is essential more research is carried out on this particular technique along with HVB in general as such intensive breathwork practices gain increasing popularity. It is important to continue to stress the message that individuals without contraindications (*cf* Fincham et al.^4^) should only practise HVB and HVBR, in settings when/where it is safe and appropriate to do so. This includes away from water and hard ground, and any situation where fainting/syncope could prove dangerous. Leaders of HVB workshops should provide thorough informed consent to potential, willing participants.

Although non-significant, it is important to note that effect sizes were small and in the expected direction for the primary analysis of greater benefits in HVBR (in particular for levels of stress, anxiety and depression at post-intervention), which could indicate possible smaller effects than expected. However, would these be clinically meaningful? It is not uncommon to find small effects such as these in studies testing unguided digital meditation and mindfulness-based interventions compared to active controls^33,34^. Therefore, there is a possibility that, whilst being the largest HVBR study to date, we may not be powered to detect very small effects of less than 0.20. If true, then a remote unguided HVBR intervention such as this delivered at scale could arguably translate into significant effects at a wider large population-level.

### Strengths

Our blinded RCT offers a methodologically robust evaluation of HVBR and is the largest trial on this type of HVB to date, with a sample balanced for gender. The practices were masked and concealed effectively, hard to achieve due to the nature of breathwork^35^. The comparator was viewed as equally credible as the intervention, offering comparable expectation of therapeutic benefit. Additionally, adherence was analogous across both groups along with overall positive sentiment, reinforcing the comparator’s plausibility. Retention rates were high (the primary timepoint had over 90% data completeness) and we included a follow-up, which other HVBR studies have not done. In addition to trait measures of mental health and wellbeing, we included both state measures of positive and negative affect in an attempt to capture any transient effects and provide a larger picture around HVBR’s effect profile. Lastly, we carefully assessed subjective adverse effects pertaining to tolerability of HVBR in terms of acute short-term side effects and long-term lasting negative effects, something lacking in HVB research^2^, and clinical research more generally from meditation^36^ to psychedelics^37^.

### Limitations

Our null findings (that outcomes for the intervention and control groups did not differ statistically) may be due to features specific to this study. For example, it may be our choice of population (i.e., young, healthy, and not highly stressed at baseline), intervention (i.e., too little, without context, not used correctly), comparator (i.e., too active), or outcomes (i.e., subjective) that could have masked or reduced the potential effects of HVBR. There may have been differences in positive emotional wellbeing, which we did not have targeted measures of. For purposes of clarity, the drawbacks of our study and methods used are discussed in terms of the *PICO* framework^38^:

#### Population

As stated above, our population being young and healthy may have meant there was less room for improvement. Accordingly, post-hoc we conducted an exploratory analysis replicating our primary analysis in participants reporting levels of stress above the normal range on the DASS subscale (15 or above). There still remained no significant group × time interaction, though the effect size more than doubled (near medium) and approached significance, (*F*(1,56) = 3.38, *p* = 0.07, *η*_*p*_^*2*^ = 0.06, *d* = 0.48). While research precisely designed to test this hypothesis is required and those with elevated baseline stress appear to be getting more benefit from HVBR in the current study, we report no robust evidence to support the idea that HVBR alone has greater efficacy than a placebo in a more symptomatic subsample. It should also be noted that participants were recruited via Prolific all of whom had been participants previously in research projects, a very specific and thus biased sample.

#### Intervention

This was a brief remote intervention and thus limits the generalisability of our findings to intensive HVB and other HVBR interventions of longer practice duration*.* Seeing as adherence was measured retrospectively using self-report, this hindered objectivity and reliability. Due to limited resources, we could not objectively track adherence (with practice engagement largely overreported in digital interventions^39^) and thus ensure compliance to the breathwork (i.e., if it was performed correctly). Real-time feedback could not be provided either due to the non-personalised delivery format. Moreover, to achieve effective and successful blinding, no context was provided around the HVBR intervention which could have diluted its effects significantly. At the expense of full automation of breathwork practices future research could deliver first sessions online to assess whether the breathwork is completed correctly, though this would require greater resources. Alternatively, to at least track breath rate, the use of remote wearables could monitor adherence to the breathwork practice both within session and across the course of the study.

#### Comparator

One possibility is that we may have in fact created a comparator intervention with active, beneficial effects, an idea previously explored in our recent slow-breathwork trial^13^. In our best attempts to avoid this, we ensured mouth breathing (without explicit instructions to breathe nasally and ‘diaphragmatically’ since these two modalities may confer their own benefit^16,40^) in the comparator group instructions. The respiratory rate was also increased from 12b/min (the lower bound of average respiratory rate) to 15b/min (what we deemed a more neutral rate). This makes it less plausible that the comparator was an effective intervention in its own right beyond its placebo effects. It could also be the rhythm we used in both the slow breathwork and HVBR studies, which is itself a conscious connected-like pattern (no pauses or holds in between inhales and exhales), had beneficial effects in its own right, though CCB is usually performed much faster^4^. Findings from uncontrolled studies are suggesting that intensive CCB can elicit altered states of consciousness phenomenologically comparable to those induced via psychedelics, and such experiences may improve subjective wellbeing and depression^15,41^. Rate aside, it might have been this conscious connected pattern (inhale:exhale) which formed an active ingredient which has inadvertently been present in the control condition and had effects equivalent in magnitude, but perhaps different in mechanisms, from HVBR, i.e., cognitive-attentional related, as a normal breathing rate should not create physiological stress in the same way as HVBR (though this may have been the case for some given the short-term side effect profile).

#### Outcomes

Following on from this, we did not have objective measures confirming control participants were normocapnic. Only respiratory monitoring data could confirm whether participants actually had different breathing patterns, thus we need to be cautious in our interpretations. The very small number of hyperventilation-related side effects suggest some briefly induced respiratory alkalosis. Pacing breathing at a given respiratory rate within the normal range does not guarantee normo-ventilating, as the resulting rate could be much lower or higher than individual resting baseline respiratory rate but also people might vary ventilation depth and therefore go into hypo- or hyper-ventilation. Moreover, absence of objective measures meant safety data relating to the most relevant risks (i.e., cardiovascular) could not be collected. Finally, if the mechanisms of HVBR are more physiological/hormonal in terms of creating objective high stress/arousal states, our outcome measures may not have been sensitive enough to capture its subjective effects. Due to highly limited resources this was not feasible. However, there is a case that mechanistic effects are indeed subjective and underpinned by attentional control. Akin to the same mechanisms of mindfulness interventions^42^, this could be because ‘conscious’ breathing reinforces present moment attention, thereby inhibiting mind wandering, rumination and worry. In other words, the control group engaged their attention for 20 min in conscious breathing, thus giving them a break from daily stress patterns such as rumination and worry.

### Recommendations

The findings of the present study do not support the use of brief, remote HVBR practice for the purpose of stress reduction in otherwise healthy, young adults. Whilst we separated a HVB component from most other elements using a reductionistic approach that is scientifically informative and useful, it is important to note that this is not a trial of HVB practices more generally. The study was carefully designed but only allowed us to measure a specific component of breathwork (i.e., increased respiratory rhythm and depth), at the expense of various other aspects of HVB which have been claimed to play a key therapeutic role^41^. Though it appears that the breathing pace in the brief, remotely delivered HVBR here did not matter, this might indicate that longer HVB techniques which induce more profound subjective changes and/or the integration with other modalities frequently used (i.e., psychological priming and bodywork), are essential aspects required to form an effective HVB intervention. Future studies can measure phenomenology such as mystical experiences, mindfulness and attentional absorption, along with natural baseline respiration rates to better match control breathing rates. Another very recent and elegant preprint with over 60 participants has suggested that accessory, contextual elements such as group/communal settings do somewhat support the emergence of altered states of consciousness induced by HVB^41^. Along with objective safety measures, more research is needed into the set and setting surrounding HVB, including context, priming and preparation in addition to sharing and integration. It may be the case that these rituals performed before and after are just as, if not more, important than the fast-breathing aspect which may be simply acting as a catalyst for change.

We hope the scientific community can build upon the strengths, and improve on the weaknesses, of our study here to inform both breathwork research and practice going forwards. Further research which clarifies and corrects any of the limitations highlighted above will move the needle. It is also our hope that contemplative psychological science research in general can benefit from our study methods used. For example, this could help further parse potential mechanisms behind mindfulness meditation techniques. Based on our findings for both slow breathwork, and now HVBR, one possibility is that, regardless of the breathing rate, the mere act of intentional, regularly paced breathing might provide beneficial effects (‘conscious breathing’). This could be due to, for example, the breath serving as a focal point for present-moment attention, counteracting negative perseverative thinking processes such as rumination or worry that are known to exacerbate depression and anxiety^42^. Future research should test these possibilities comparing ‘conscious’ breathing (but explicitly not mindfulness meditation, i.e., without the contextual qualities frequently provided in these practices) to alternative comparators which place attention elsewhere and do not control the breath, which our lab now intends to do. This will likely involve multi-arm component studies using attentional oscillators (i.e., sound, sensation) similar in rhythm to breathing (but without breath control) to account for active effects of breath regulation, and using outcomes pertaining to attention, worry and rumination. Ultimately, we hope the reductionist approach used here informs future research on HVB practices that are supported by growing use and popularity.

### Supplementary Information

Supplementary Figures.

## Data Availability

The datasets used and/or analysed during the current study available from the corresponding author on reasonable request.
