# Supplementary material for: Effects of brief remote high ventilation breathwork with retention on mental health and wellbeing: a randomised placebo-controlled trial
Source: Sci Rep. 2024 Jul 23;14:16893. doi: 10.1038/s41598-024-64254-7 (PMC11266346; doi:10.1038/s41598-024-64254-7)
Supplement: Supplementary file 1 — Supplementary Figures. [file 41598_2024_64254_MOESM1_ESM.docx]

**Supplement**

**Effects of brief remote** **high ventilation breathwork with retention on mental health and wellbeing: A randomised placebo-controlled trial**

Guy W. Fincham^1,2*^, Elissa Epel^3*^, Alessandro Colasanti^2,4^, Clara Strauss^1,4^, Kate Cavanagh^1,4^

^1^ School of Psychology, University of Sussex, Brighton, UK

^2^ Brighton & Sussex Medical School, University of Sussex, UK

^3^ Department of Psychiatry & Behavioral Sciences, University of California San Francisco, US

^4^ Sussex Partnership NHS Foundation Trust, UK

*Corresponding author

[g.fincham@sussex.ac.uk](mailto:g.fincham@sussex.ac.uk)

[elissa.epel@ucsf.edu](mailto:elissa.epel@ucsf.edu)

Downloadable audio links for: [Intervention](https://youtu.be/yTqthtuzVzE) and [Control](https://www.youtube.com/watch?v=k1V2Y_fzyYc)


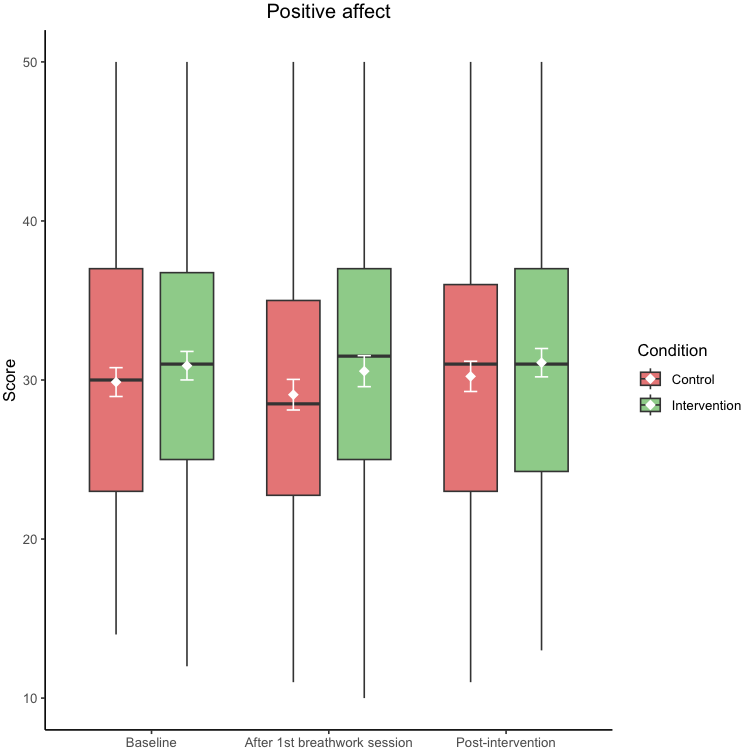


**Figure S1.** Score distribution for secondary outcome positive affect at baseline, after first breathwork session, and post-intervention for both intervention (green) and control (red) conditions. Means (±95% CI error bars) in white and medians are middle lines within boxes, with higher scores inferring greater positive affect (score range 10-50). Boxplots also display quartiles. Figure produced using *R* (version 4.3.2).


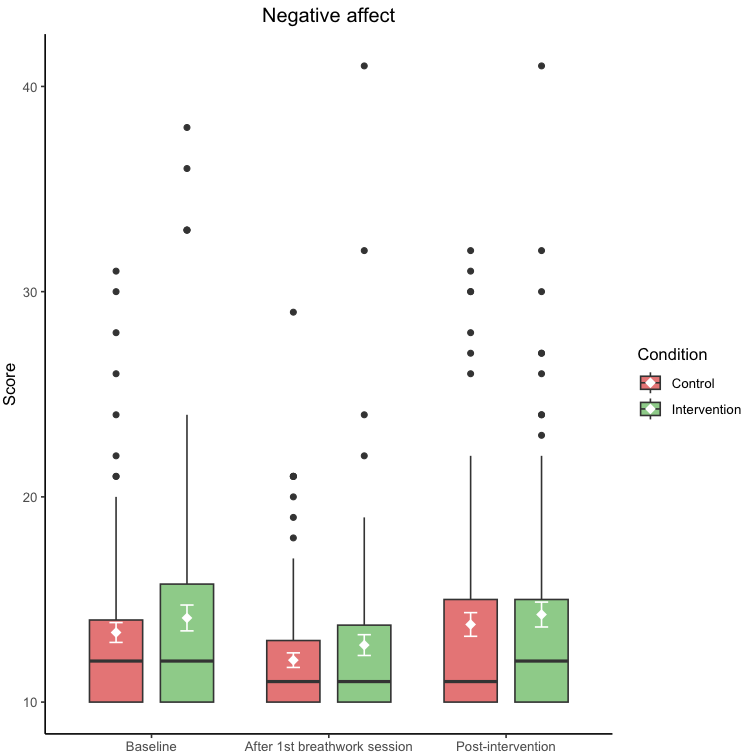


**Figure S2.** Score distribution for secondary outcome negative affect at baseline, after first breathwork session, and post-intervention for both intervention (green) and control (red) conditions. Means (±95% CI error bars) in white and medians are middle lines within boxes, with higher scores inferring greater negative affect (score range 10-50). Boxplots also display quartiles and circles represent outliers. Figure produced using *R* (version 4.3.2).


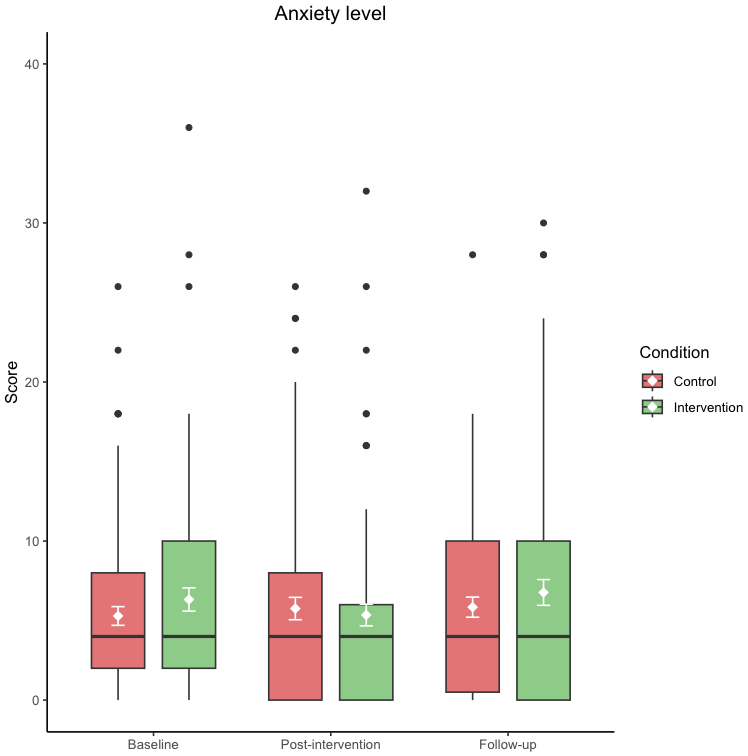


**Figure S3.** Score distribution for secondary outcome anxiety across all timepoints (pre-post-follow-up) for both intervention (green) and control (red) conditions. Means (±95% CI error bars) in white and medians are middle lines within boxes, with higher scores inferring greater anxiety (score range 0–42). Boxplots also display quartiles and circles represent outliers. Figure produced using *R* (version 4.3.2).


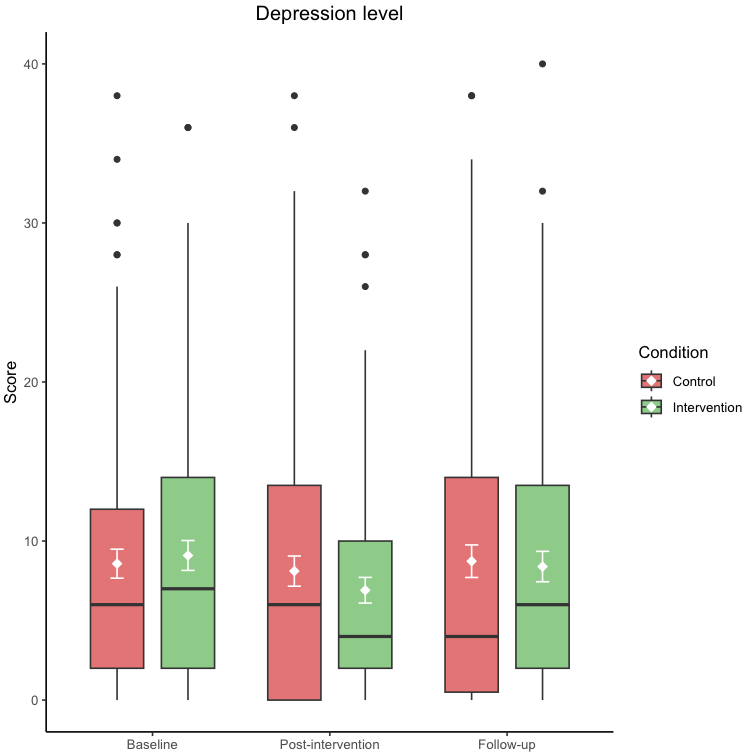


**Figure S4.** Score distribution for secondary outcome depression across all timepoints (pre-post-follow-up) for both intervention (green) and control (red) conditions. Means (±95% CI error bars) in white and medians are middle lines within boxes, with higher scores inferring greater depression (score range 0–42). Boxplots also display quartiles and circles represent outliers. Figure produced using *R* (version 4.3.2).


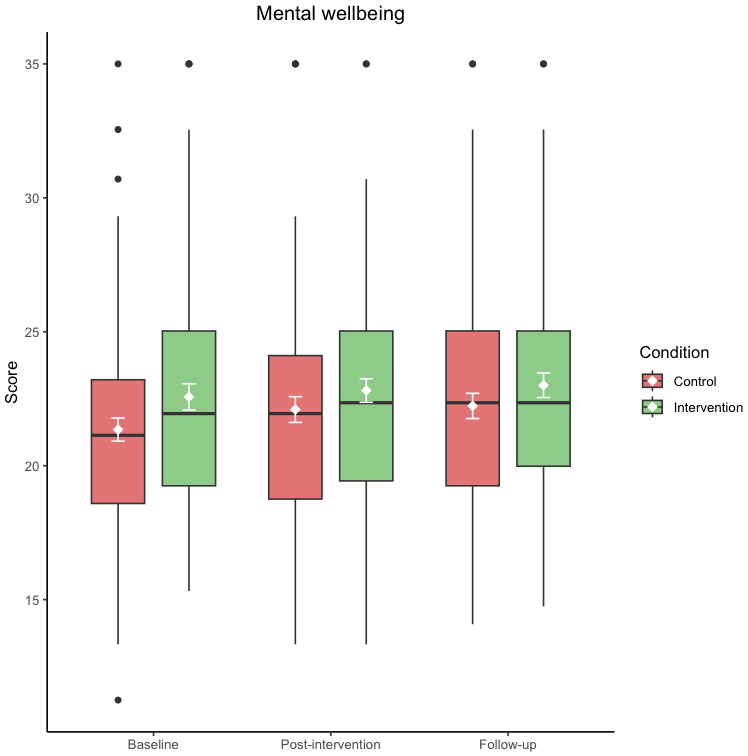


**Figure S5.** Score distribution for secondary outcome mental wellbeing across all timepoints (pre-post-follow-up) for both intervention (green) and control (red) conditions. Means (±95% CI error bars) in white and medians are middle lines within boxes, with higher scores inferring greater mental wellbeing (score range 7-35). Boxplots also display quartiles and circles represent outliers. Figure produced using *R* (version 4.3.2).


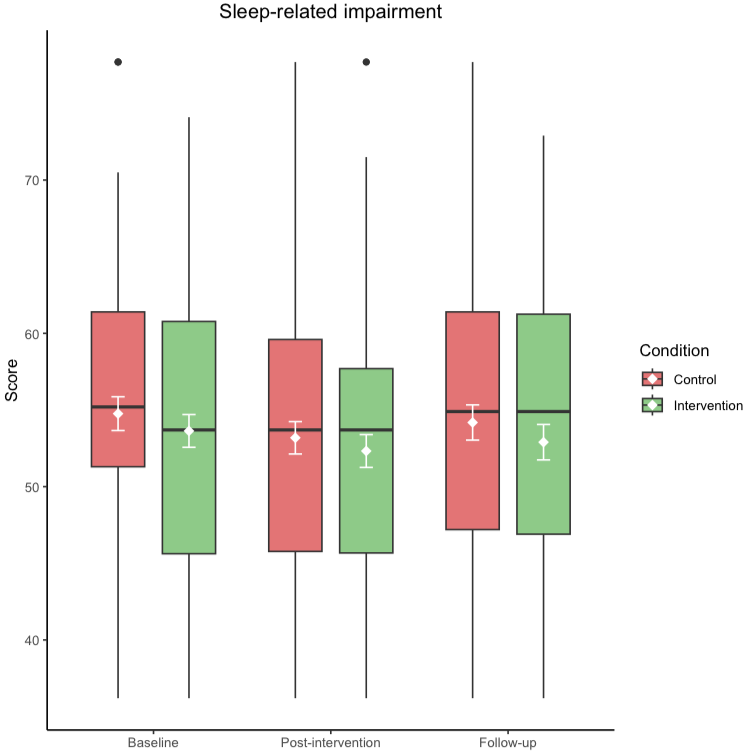


**Figure S6.** Score distribution for secondary outcome sleep-related impairment across all timepoints (pre-post-follow-up) for both intervention (green) and control (red) conditions. Means (±95% CI error bars) in white and medians are middle lines within boxes, with higher scores inferring greater sleep-related impairment (score range 30.5-77.5). Boxplots also display quartiles and circles represent outliers. Figure produced using *R* (version 4.3.2).
